# 

**Published:** 1893-01-07

**Authors:** 


**Notices of Births, Deaths, and Marriages,** are inserted in this column at a charge of 2s. 6d. for an announcement not exceeding four lines.

**DEATH.**

On the 29th inst., at the Royal Infirmary, Liverpool, in the 53rd year of her age, Miss EMMELINE STAINS, for over 11 years Lady Superintendent of the Royal Infirmary, and of the Liverpool Training School and Home for Nurses, Dover Street; after 25 years of invaluable nursing work.

### NOTICE TO CORRESPONDENTS.

All MS., Letters, Books for Review, and other matters intended for the Editor should be addressed THE EDITOR, THE LODGE, PORCHESTER SQUARE, LONDON, W.

The Editor cannot undertake to return rejected M.S., even when accompanied by stamped directed envelope.

**Notice to Advertisers and Subscribers.**

All Advertisements, Orders for Copies of THE HOSPITAL, and Business Communications should be addressed to The Publisher, *not to the Editor*, THE HOSPITAL, 140, Strand, W.C.

The Cost of Subscription, post free, is as follows:—Per week, 2½d.; per quarter, 2s. 6d.; per half-year, 5s.; per annum, 8s. 6d. Foreign:—Per annum, 10s. 8d.; payable, in all cases, in advance.

**"Burdett's Hospital Annual," 1893.**

Fourth year of publication. 700 pp. Boards, gilt lettering. A most complete guide to British, Colonial, Indian, and American Hospitals, Dispensaries, Nursing and Convalescent Institutions, and Asylums. Price 5s. Published by THE SCIENTIFIC PRESS (Limited), 140, Strand, W.C.

**NOTICE.—The Index for Vol. XII. (ending September 1892) is on sale at the Office of this Journal, price 2½d., post free.**

### THE STUDENT OF MEDICINE.

[All communications for this Supplement should be addressed to THE EDITOR of THE HOSPITAL, at 140, Strand, with the words, "Students' Column," written in the left hand top corner of the envelope.]

### VACANCIES.

The following vacancies are announced this week, the amount of salary in each case being given after the name of the institution:

### Resident Medical Officers.

Alnwick Infirmary. House Surgeon. Salary £120 per annum, with furnished apartments, attendance, coal, and gas. Applications to W. T. Hindmarsh, 26, Bondgate Without, Alnwick, by January 14th.

Ancoats Hospital, Manchester. Resident Junior House Surgeon. Salary £50 per annum, with board and washing. Applications to Alexander Forrest, Honorary Secretary.

Central London Ophthalmic Hospital, Gray's Inn Road, W.C. House Surgeon. Rooms, coal, and light provided. Applications to the Secretary, by January 10th.

Chichester Infirmary, Chichester. House Surgeon. Salary £80 per annum, with board, lodging, and washing. Applications to E. E. Street, Secretary, by March 1st.

Derbyshire Royal Infirmary. Resident Assistant House Surgeon. Duly qualified and registered. £10 for six months, £25 for second six months. Applications must be received by January 19th. Separate apartments, board, and washing.

Hospital for Consumption and Diseases of the Chest, Brompton. House Physicians. Applications to the Secretary by January 12th.

Killarney District Lunatic Asylum. Assistant Medical Officer. Salary £100 per annum, with allowances valued at £100 yearly. Candidates must be unmarried, and not over 30. Applications to Dr. L. T. Griffin, Resident Medical Superintendent. Election on January 19th.

Manchester Royal Infirmary. Resident Medical Officer for the Fever Hospital at Monsall; not less than 25 years of age, doubly qualified. Salary £250 per annum, with board and residence. Applications to the Chairman of the Board by January 7th.

National Hospital for the Paralysed and Epileptic (Albert Memorial), Queen Square, Bloomsbury, W.C. House Physician, doubly qualified. Salary £100 per annum, with board and apartments. Applications to B. Burford Rawlings, Secretary and General Director, by January 5th.

North Staffordshire Infirmary and Eye Hospital, Hartshill, Stoke-on-Trent. House Surgeon; doubly qualified. Salary £120 per annum, increasing £10 yearly, with furnished apartments, board, and washing. Applications to the Secretary by January 21st.

Parish of Applecross. Ross-shire. Medical Officer for a Division. Emoluments from public sources, £105 per annum, with house and garden free. Applications to Mr. D. Bain, Applecross, by Jan. 7th.

Victoria Hospital for Sick Children, Queen's Road, Chelsea, S.W. House Physician to the In-patients; doubly qualified. Honorarium £50 per annum, with board and lodging. Applications to the Secretary by January 14th.

Victoria Hospital for Sick Children, Queen's Road, Chelsea, S.W. House Surgeon to the In-patients; doubly qualified. Honorarium £50 per annum, with board and lodging. Applications to the Secretary by January 14th.

West Herts Infirmary, Hemel Hempstead. House Surgeon and Dispenser, doubly qualified, unmarried. Salary £100 per annum, with board, furnished rooms, light, fire, attendance, and washing. Applications to the Secretary by January 31st.

### Non-Resident Medical Officers.

Hostel of St. Luke, Devonshire Street, W. Honorary Visiting Medical Officer. Applications to the Secretary, Hostel of St. Luke, Church House, Westminster, by January 7th.

King's College, London. Assistant Physician for Diseases of the Throat at King's College Hospital. Applications to J. W. Cunningham, Secretary.

Stratford-on-Avon Rural Sanitary Authority. Medical Officer of Health. Salary £110 per annum. Applications to John Charles Warden, 9, Guild Street, Stratford-on-Avon, by January 21st.

Stratford-on-Avon Urban Sanitary Authority. Medical Officer of Health. Salary £40 per annum. Applications to Thomas Hunt, Town Clerk, Municipal Office, Stratford-on-Avon, by January 2nd.

University College of South Wales and Monmouthshire, Cardiff. A Professor of Anatomy, and a Professor of Physiology. Stipend in each case £350 per annum. Applications to Ivor James, Registrar, by February 10th.

University of Edinburgh. Examiner in each of the following subjects: (1) Anatomy, (2) Chemistry and Laboratory Work for the First B.Sc. Examination in Public Health, (3) Midwifery, (4) Practice of Physic, and (5) Botany. The salary in each of the departments Nos. 1, 3, 4, and 5 is £75 a year, and in No. 2 £90 a year, with an allowance of £10 per annum for travelling and other expenses in the case of Examiners not resident in Edinburgh or the immediate neighbourhood. Applications to M. C. Taylor, Secretary, by January 9th.

Salary £80 per annum, with Board, Lodging, and Washing.

### THE STUDENT OF MEDICINE—(*continued*).

University of Glasgow. Assistant Examiner in Zoology. Salary £30 per annum. Applications to the Secretary of the Court, Mr. Alan E. Clapperton, West Regent Street, Glasgow, by January 10th.

### PASS LISTS.

### University of London.

1892.—M.D. EXAMINATION.

MEDICINE.—H. G. Adamson, St. Bartholomew's Hospital; W. H. Alchin, Westminster Hospital; L. W. Andrews, St. Bartholomew's Hospital; H. W. C. Austen, B.S., St. Bartholomew's Hospital; Sir H. R. Beevor, Bart., King's College; Letitia C. Bernard, London School of Medicine and Royal Free Hospital; Frances M. D. Berry, London School of Medicine and Royal Free Hospital; R. C. Bowman, Manchester Royal Infirmary: D. Brown, B.Sc., London Hospital; L. T. F. Bryett, King's College; G. S. Buchanan, B.S., B.Sc., St. Bartholomew's Hospital; A. W. Burrell, London Hospital; F. G. Bushnell, University College; †H. A. Caley, St. Mary's Hospital; J. St. T. Clarke, Guy's Hospital; C. Coles (gold medal), St. Bartholomew's Hospital; W. S. Colman, University of Edinburgh and University College, London; S. B. Cook, St. Thomas's Hospital; G. H. Cooke, Owens College and Manchester Royal Infirmary; H. Corner, London Hospital; H. J. Curtis, B.S., University College; †P. R. Dodwell, University College; D. Drew, B.S., University College; H. H. Fisher, St. Bartholomew's Hospital; †A. E. Giles, B.Sc., Owens College; D. R. Green, B.S., University College; A. Griffith, University College; R. T. Hewlett, King's College; T. W. Hinds, University College; A. D. P. Hodges, London Hospital; H. Horrocks, B.Sc., Owens College and Manchester Royal Infirmary; E. V. Hugo, B.S., St. Bartholomew's Hospital; W. R. Jordan, Queen's and Mason Colleges, Birmingham; A. A. Kanthack, B.S., B.A., B.Sc., Liverpool Royal Infirmary, St. Bartholomew's Hospital, and University Cambridge; H. B. Kitchin, University College; R. E. S. Krohn, University College; A. W. W. Lea, B.S., Owens College and Manchester Royal Infirmary; P. Leech, B.S., Owens College and Manchester Royal Infirmary; J. S. McGowan, B.S., B.Sc., Owens College and Manchester Royal Infirmary; J. J. Macgregor, St. Bartholomew's Hospital; C. P. Oliver, Charing Cross Hospital; J. E. Paul, University College; H. J. M. Playfair, King's College; †H. M. Richards, B.S., University College; R. W. Richards, St. Bartholomew's Hospital; L. Roberts, St. Bartholomew's Hospital; R. G. Rows, University College; E. A. Sadler, Birmingham Medical School; H. Sharman, University College; G. A. Simmons, B.S., St Mary's Hospital; E. N. Smith, University College; C. R. Stevens, B.S., St. Bartholomew's Hospital; T. G. Stevens, B.S., Guy's Hospital; †W. M. Stevens, University College; W. F. Umney, St. Thomas's Hospital; J. Wilkie, B.Sc., St. Bartholomew's Hospital; J. P. Williams, Owens College and Manchester Royal Infirmary; A. S. Wohlmann, B.S., Guy's Hospital; Emily E. Wood, London School of Medicine and Royal Free Hospital; T. J. Wood, University College.

† Obtained the number of marks qualifying for the Gold Medal.

STATE MEDICINE.—A. E. Permewan, M.D., University College; R. T. Sisley, M.D., St. George's, Guy's, University College, and Vienna; T. Thomson, Universities of Aberdeen and Edinburgh; F. Tratman, Bristol, and London Hospital.

B.S. EXAMINATION.

PASS LIST.

FIRST DIVISION.—H. A. Ballance, University College; W. Bligh, Guy's Hospital; J. L. Firth, M.D., University College; E. T. E. Hamilton, B.Sc., Guy's Hospital; J. W. F. Jewell, Guy's Hospital; W. B. Jones, St. Bartholomew's Hospital; T. D. Lister, Guy's Hospital; C. S. Pantin, Guy's Hospital; E. P. Paton, M.D., St. Bartholomew's Hospital; C. H. Preston, Owens, Manchester Royal Infirmary, and London Hospital; L. Rogers, St. Mary's Hospital; H. S. Sandifer, King's College; G. A. Stephens, B.Sc., University College; T. R. Taylor, B.Sc., Guy's Hospital; F. W. Wesley, University College.

SECOND DIVISION.—C. Addison, St. Bartholomew's Hospital; H. S. Ballance, King's College; A. S. Blackwell, B.Sc., St. Bartholomew's Hospital; C. R. Box, B.Sc., St. Thomas's Hospital; C. C. Chidell, University College; F. J. Coleman, Guy's Hospital; A. M. Daldy, Guy's Hospital; C. C. Elliott, Guy's Hospital; S. G. Floyd, Guy's Hospital; C. J. Harrison, University College; M. L. Hepburn, St. Bartholomew's Hospital; J. Jones, University College; H. T. Parker, St. Bartholomew's Hospital; W. J. Procter, London Hospital; A. J. Sharp, Guy's Hospital; A. W. Sheen, Guy's Hospital; C. E. Wheeler, B.Sc., St. Bartholomew's Hospital; S. L. B. Wilks, The Yorkshire College.

EXAMINATION FOR HONOURS.—SURGERY.

FIRST CLASS.—E. P. Paton, M.D. (Scholarship and Gold Medal), St. Bartholomew's Hospital; H. A. Ballance (Gold Medal), University College; †H. S. Sandifer, King's College; C. S. Pantin, Guy's Hospital; E. T. E. Hamilton, B.Sc., Guy's Hospital; T. R. Taylor, B.Sc., Guy's Hospital.

SECOND CLASS.—J. L. Firth, M.D., University College; L. Rogers, St. Mary's Hospital; W. B. Jones, St. Bartholomew's Hospital; C. H. Preston, Owens, Manchester Royal Infirmary, and London Hospital; T. D. Lister, Guy's Hospital.

† (Worthy of Medal.)

THIRD CLASS.—F. J. Coleman, Guy's Hospital, A. W. Sheen, Guy's Hospital, and C. E. Wheeler, St. Bartholomew's Hospital (equal); J. W. F. Jewell, Guy's Hospital.

M.S. EXAMINATION.

PASS LIST.

T. B. P. Davies, Guy's Hospital (gold medal); F. W. Hall, M.D., Guy's Hospital; R. Pickard, St. Bartholomew's Hospital; †W. G. Rogers, Guy's Hospital.

(† Obtained the number of marks qualifying for the gold medal.)

